# Modulation of Colorectal Cancer Risk by Polymorphisms in 51Gln/His, 64Ile/Val, and 148Asp/Glu of APEX Gene; 23Gly/Ala of XPA Gene; and 689Ser/Arg of ERCC4 Gene

**DOI:** 10.1155/2017/3840243

**Published:** 2017-03-12

**Authors:** L. Dziki, A. Dziki, M. Mik, I. Majsterek, J. Kabzinski

**Affiliations:** ^1^Department of General and Colorectal Surgery, Medical University of Lodz, Lodz, Poland; ^2^Department of Clinical Chemistry and Biochemistry, Medical University of Lodz, Lodz, Poland

## Abstract

Polymorphisms in DNA repair genes may affect the activity of the BER (base excision repair) and NER (nucleotide excision repair) systems. Using DNA isolated from blood taken from patients (*n* = 312) and a control group (*n* = 320) with CRC, we have analyzed the polymorphisms of selected DNA repair genes and we have demonstrated that genotypes 51Gln/His and 148Asp/Glu of APEX gene and 23Gly/Ala of XPA gene may increase the risk of colorectal cancer. At the same time analyzing the gene-gene interactions, we suggest the thesis that the main factor to be considered when analyzing the impact of polymorphisms on the risk of malignant transformation should be intergenic interactions. Moreover, we are suggesting that some polymorphisms may have impact not only on the malignant transformation but also on the stage of the tumor.

## 1. Introduction

Currently, we are observing an increase of the incidence of colorectal cancer (CRC). In 2012, according to GLOBCAN, there were 1360000 new CRC cases, which with 9.7% made it the third most common cancer after lung and breast cancers [1, 2]. While causes of CRC remain unknown, it is estimated that about 20% of cancer cases are familial and approximately 3% are caused by mutations of strongly predisposed genes [3, 4]. Studies have shown that individual predispositions for developing this cancer may depend on genetic changes, including changes in genes involved in the process of DNA repair, which is responsible for dealing with DNA damages [5–7]. Several single-nucleotide polymorphisms (SNPs) have been associated with colorectal cancer susceptibility; most of them are part of mismatch DNA repair system (MMR) [8–10]. However, besides MMR system in mammalian cells, there are three more basic mechanisms of DNA repair: BER (base excision repair), NER (nucleotide excision repair), and DSB (double-strand brakes), which are currently under strong investigation in terms of connection with an increased risk of colorectal cancer [11–13].

In this paper, we study the selected polymorphisms of nucleotide excision repair (NER) and base excision repair (BER) pathways and their impact on modulating risk of colorectal cancer occurrence. Among the known polymorphisms of the DNA repair genes, the polymorphisms of *ERCC4* and *XPA* genes from NER pathway have been repeatedly studied as potentially connected with susceptibility to the occurrence of various cancers [14–17]. NER is a particularly important excision mechanism that removes DNA damage induced by ultraviolet light (UV). UV DNA damage results in bulky DNA adducts—these adducts are mostly thymine dimers and 6,4-photoproducts. The importance of NER is evidenced by the severe human diseases that result from in-born genetic mutations of NER proteins such as xeroderma pigmentosum and Cockayne's syndrome [18, 19]. The second studied pathway—BER—is a DNA repair system that operates on small lesions such as oxidized or reduced bases. A single damaged base is removed by base-specific DNA glycosylases and apurinic/apyrimidinic sites are created (that can occur also by spontaneous hydrolysis or by DNA damaging agents). AP sites are premutagenic lesions that can prevent normal DNA replication and therefore need to be identified and repaired. Whole process is initiated by the major AP endonuclease in human cells coded by *APEX* gene, whose polymorphisms have been so far connected to several types of cancer [20–22]. Moreover, our goal was to evaluate the mutual action of those two DNA repair systems on modulating CRC risk.

## 2. Materials and Methods

DNA for genotyping was isolated from lymphocytes of the peripheral blood. The blood samples were taken from 312 unrelated patients hospitalized in the Military Medical Academy University Teaching Hospital-Central Veterans' Hospital in Lodz. Each patient had histopathologically confirmed colorectal cancer. The studied group included 178 men and 134 women (average age 63 years ± 8 years). The stage of the tumors was established according to the TNM scale. The control group included 320 individuals not diagnosed with cancer and with ages corresponding to the age of the studied group (*p* < 0.05). Permission to conduct research was granted by the Bioethics Committee of the Medical University of Lodz.

DNA isolation was carried out with a commercial kit QIAamp DNA Blood Mini Kit for isolation of high-molecular weight DNA (Qiagen).

The occurrence of polymorphic variants in 51Gln/His, 64Ile/Val, and 148Asp/Glu of *APEX* gene; 23Gly/Ala of *XPA* gene; and 689Ser/Arg of *ERCC4* gene was studied with the TaqMan technique. Briefly, 25 *μ*l of reaction mixture was used for analysis, containing 1 *μ*l of genomic DNA solution, 1 *μ*l of probes designed specifically for each polymorphism, 13 *μ*l of premix with polymerase, and 10 *μ*l of water. The PCR reaction was performed in a Stratagene Mx3005P real-time PCR thermocycler. The RS numbers for polymorphisms and thermal conditions of reaction are shown in Table 1. For 10% of the randomly selected samples, genotyping was repeated to confirm reproducibility. Cases and controls were genotyped randomly and researchers were blinded to the case/control status during genotyping.

## 3. Results

### 3.1. Genotyping

The genotyping results indicate that Gln/His genotype of 51Gln/His polymorphism of *APEX* gene (Table 2) increases the risk of CRC (OR 1.706 (1.174–2.480); *p* = 0.005). The same effect was observed in the case of Asp/Glu genotype (Table 3) of 148Asp/Glu polymorphism of *APEX* gene (OR 2.588 (1.736–3.859); *p* < 0.0001) and Gly/Ala genotype (Table 4) of 23Gly/Ala polymorphism of *XPA* gene (OR 5.373 (3.418–8.446); *p* < 0.0001). We did not find any significant influence of 64Ile/Val polymorphism of APEX gene (Table 5) and 689Ser/Arg polymorphism of ERCC4 gene (Table 6) on modulation of CRC risk.

### 3.2. Gene-Gene Interactions

In order to investigate the interaction of the polymorphisms of the studied genes and to evaluate their mutual influence on the risk of colorectal cancer, gene-gene interactions were analyzed. Results are presented in Table 7; only pairs that modulate the risk at a statistically significant level are shown. For the full set of results showing all pairs of gene-gene interactions, please refer to the tables in Supplementary Material available online at https://doi.org/10.1155/2017/3840243. It has been revealed that genotype pair Gln/His-Val/Val for 51Gln/His *APEX*-64Ile/Val *APEX* increases the risk of CRC. For 51Gln/His *APEX*-148Asp/Glu *APEX*, we can observe increased risk in the case of Gln/His-Asp/Glu pair, but at the same time, coincidence of genotypes Gln/His-Glu/Glu and His/His-Asp/Asp decreases the risk. For pair 51Gln/His *APEX*-23Gly/Ala *XPA*, we observed increased risk in the case of genotypes Gln/His-Gly/Ala and His/His-Gly/Ala and decreased risk for Gln/His-Gly/Gly. Moreover, increased risk of colorectal cancer was revealed for pairs Gln/His-Arg/Arg (51Gln/His *APEX*-689Ser/Arg *ERCC4*), Val/Val-Asp/Glu (64Ile/Val *APEX*-148Asp/Glu *APEX*), Val/Val-Gly/Ala (64Ile/Val *APEX*-23Gly/Ala *XPA*), and Asp/Glu-Gly/Ala (148Asp/Glu *APEX*-23Gly/Ala *XPA*); while at the same time, risk was decreased for pairs Ile/Val-Asp/Asp (64Ile/Val *APEX*-148Asp/Glu *APEX*), Ile/Val-Gly/Gly, and Ile/Val-Ala/Ala (64Ile/Val *APEX*-23Gly/Ala *XPA*) as well as Asp/Asp-Gly/Ala and Asp/Glu-Gly/Gly (148Asp/Glu *APEX*-23Gly/Ala *XPA*). In addition, worth noticing is the major impact of Asp/Glu genotype of 148Asp/Glu *APEX* gene when paired with all genotypes of 689Ser/Arg *ERCC4*, and similarly, Gly/Ala genotype again paired with all genotypes of 689Ser/Arg *ERCC4*.

### 3.3. Influence on Tumor Progression

In addition, we wanted to investigate potential correlation of our results with clinical data; therefore impact of presence of studied polymorphisms on progression of stage of tumor was tested, by a correlation of the distribution of genotypes with the state of tumor by the American Joint Committee on Cancer classification. Results are presented in Table 8. We found that 148Asp/Glu polymorphism of *APEX* gene and 23Gly/Ala polymorphism of XPA gene are increasing the risk of cancer in the second degree of advancement in relation to the first degree.

## 4. Discussion

All cells of the human body are constantly exposed to damaging agents, which can cause changes in the DNA. These changes, if not repaired, may lie at the basis of the process of carcinogenesis. To cope with those damages, the human body has developed a number of DNA repair mechanisms, including BER and NER systems. One of the key elements of BER is *APEX* gene product—class II AP endonuclease. Endonuclease cleaves the phosphodiester backbone 5′ to the AP site, thereby initiating a repair process [23]. Polymorphisms in *APEX* gene have been for a long time a subject of interest in the area of modulating risk of malignant transformation and many of them have been connected to several types of cancers such as lung cancer, breast cancer, or bladder cancer [24–28]. In the case of colorectal cancer, it has been estimated that *APEX* Asp148Glu is involved in increasing CRC risk [21, 29] which is consistent with our results. However, some researchers suggest that there is no association between increased cancer risk and the *APEX* Asp148Glu polymorphisms [20] or even that its occurrence decreases the risk—Brevik et al. report that carriers of the *APEX* codon 51 Gln/His genotype had a reduced CRC risk compared with carriers of the Gln/Gln genotype [30]. In contrast to these reports, we in our study found that Gln/His genotype increases the CRC risk. Similar differences can be observed in the case of NER system genes studied by us—23Gly/Ala of *XPA* gene is suggested to have no influence on risk of colorectal cancer [31, 32], while our results indicate the opposite (odds ratio (OR) 5.373 (3.418–8.446); *p* < 0.0001; Table 4). We believe that the cause of such divergence may lie in different selections of the study group (presented studies were carried out on the Japanese and Turkish populations while our research was on the Polish population) as well as bias in the selection because of the diet (eating red meat) and smoking tobacco, since it has been proven that ethnic group as well as other factors have a significant impact on the modulation of the risk of particular diseases [33, 34]. However, in our opinion, the main factor that could cause individual differences in modulation of risk by the same polymorphisms is gene-gene interactions. Several studies have confirmed that the polymorphisms of individual genes can significantly change the level of risk in case of coexistence with other specific polymorphisms. This phenomenon is observed even in cases when those polymorphisms do not have a significant effect on the modulation of the cancer risk when studied without mentioned coexistence [35–39]. Therefore, in the second part of this work, we have made calculations of gene-gene interactions to test the impact of a joint action examined in earlier polymorphisms. Results are shown in Table 7. The first thing worth noting is the increased risk of CRC in the case of co-occurrence of genotype 51Gln/His of *APEX* gene with 64Val/Val of *APEX* gene when compared to risk associated only with 51Gln/His (OR 2.266 (1.120–4.585); *p* = 0.022 versus 1.706 (1.174–2.480); *p* = 0.005) and similar increased risk of CRC in the case of co-occurrence of genotype 51Gln/His of *APEX* gene with 689Arg/Arg ERCC4 (OR 2.464 (1.247–4.870); *p* = 0.009 versus 1.706 (1.174–2.480); *p* = 0.005). Similar increased risk can also be seen in the case of pairs 51Gln/His *APEX*-148Asp/Glu *APEX* and 51Gln/His *APEX*-23Gly/Ala *XPA*; however, 148Asp/Glu *APEX* and 23Gly/Ala *XPA* also considered individually increased risk of CRC. That is why we want to pay special attention to the first two pairs (51Gln/His *APEX*-64Val/Val *APEX* and 51Gln/His *APEX*-689Arg/Arg *ERCC4*) in which only 51Gln/His *APEX* increases the risk, but in the case of coexistence with the aforementioned polymorphisms, this risk becomes even greater. In our opinion, this clearly indicates a much more advanced system of impact of polymorphisms on the risk of cancer than the effect of a SNP. It can also indicate the interaction of BER and NER systems in removing damage which has been suggested by other researchers [40]. Confirmation of the thesis of common effect of polymorphisms of different genes to modulate the risk of cancer is also observed by us as protective effect in form of reducing the risk of CRC for genotype pairs (51Gln/His *APEX*-148Glu/Glu *APEX* and 51Gln/His *APEX*-23Gly/Gly *XPA*) where one of the polymorphisms previously increased the risk, so we can see a reversal of the trend. It is also worth noting that the protective effect may have a pair in which none of the polymorphisms, when considered individually, showed no previous effect on the risk of CRC increasing nor decreasing it (64Ile/Val *APEX*-23Ala/Ala *XPA* and 148Asp/Glu *APEX*-23Gly/Gly *XPA*). Finally, we want to draw attention to the fact that genotype 148Asp/Glu *APEX* which itself increases the risk of CRC (OR 2.588 (1.736–3.859); *p* < 0.0001), when considered along with any polymorphism of 689Ser/Arg *ERCC4* (Ser/Ser, Ser/Arg, or Arg/Arg), will always show a greater risk than alone (OR, resp., 2.716 (1.293–5.704), *p* = 0.007, 2.963 (1.467–5.985), *p* = 0.002, and 2.643 (1.171–5.965), *p* = 0.018). In our opinion, this confirms the thesis pronounced earlier that the gene-gene interactions are the main factor that may influence individual differences in the predisposition to the occurrence of cancer.

Given such a complicated set of factors and their mutual interactions that may cause malignant transformation, it should be also suspected that there can be whole set of various factors contributing to the progression of already formed tumor. In order to evaluate the impact of polymorphisms of analyzed genes on the progress of colorectal cancer, we have correlated the distribution of genotypes with the progress of the tumor according to the classification of the American Joint Committee on Cancer. Increased risk of CRC in the II° of advancement in relation to the I° was observed in the case of 148Asp/Glu polymorphism of *APEX* gene and 23Gly/Ala polymorphism of XPA gene (Table 8). The observed effect is consistent with the effect of the polymorphisms in the increased risk of disease onset, which may suggest the relationship between specific genotypes and initiation and promotion of carcinogenesis as well as the progression of cancer and metastasis. This is in line with our earlier studies in which we presented similar thesis [38]. In our opinion, this allows not only to identify patients with an increased risk of cancer but also to identify within the group of patients already diagnosed with the disease to predict potential tumor growth and thus allows a more comprehensive approach to treating patients.

## 5. Conclusions

Genotypes 51Gln/His and 148Asp/Glu of APEX gene and 23Gly/Ala of XPA gene may increase the risk of CRC, while polymorphisms of 64Ile/Val of APEX gene and 689Ser/Arg of ERCC4 gene have no effect on modulating the risk. At the same time, gene-gene interactions may completely change the risk level; therefore, we advocate that they should be considered as very important factors when calculating the risk factor. Moreover, polymorphisms of BER and NER systems may not only initiate malignant transformation but also be able to contribute to tumor growth. We believe that our results are promising, yet further studies are needed on this subject to establish an incontestable link between a given polymorphism and its phenotypic effect in the modulation of BER and NER activity and thus its impact on carcinogenesis.

## Supplementary Material

Table 1. The distribution of genotypes and the analysis of the odds ratio (OR) for gene-gene interactions: 51Gln/His APEX – 64Ile/Val APEX in patients with colorectal cancer (CRC) and the control group. Table 2. The distribution of genotypes and the analysis of the odds ratio (OR) for gene-gene interactions: 51Gln/His APEX – 148Asp/Glu APEX in patients with colorectal cancer (CRC) and the control group. Table 3. The distribution of genotypes and the analysis of the odds ratio (OR) for gene-gene interactions: 51Gln/His APEX – 23Gly/Ala XPA in patients with colorectal cancer (CRC) and the control group. Table 4. The distribution of genotypes and the analysis of the odds ratio (OR) for gene-gene interactions: 51Gln/His APEX – 689Ser/Arg ERCC4 in patients with colorectal cancer (CRC) and the control group. Table 5. The distribution of genotypes and the analysis of the odds ratio (OR) for gene-gene interactions: 64Ile/Val APEX – 148Asp/Glu APEX in patients with colorectal cancer (CRC) and the control group. Table 6. The distribution of genotypes and the analysis of the odds ratio (OR) for gene-gene interactions: 64Ile/Val APEX – 23Gly/Ala XPA in patients with colorectal cancer (CRC) and the control group. Table 7. The distribution of genotypes and the analysis of the odds ratio (OR) for gene-gene interactions: 64Ile/Val APEX – 689Ser/Arg ERCC4 in patients with colorectal cancer (CRC) and the control group. Table 8. The distribution of genotypes and the analysis of the odds ratio (OR) for gene-gene interactions: 148Asp/Glu APEX – 23Gly/Ala XPA in patients with colorectal cancer (CRC) and the control group. Table 9. The distribution of genotypes and the analysis of the odds ratio (OR) for gene-gene interactions: 148Asp/Glu APEX – 689Ser/Arg ERCC4 in patients with colorectal cancer (CRC) and the control group. Table 10. The distribution of genotypes and the analysis of the odds ratio (OR) for gene-gene interactions: 23Gly/Ala XPA – 689Ser/Arg ERCC4 in patients with colorectal cancer (CRC) and the control group.

## Figures and Tables

**Table 1:** The refSNP and thermal conditions used in the PCR reaction.

Gene	*APEX*	*APEX*	*APEX*	*XPA*	ERCC4
Polymorphism	51Gln/His	64Ile/Val	148Asp/Glu	23Gly/Ala	689Ser/Arg

refSNP	rs1048945	rs2307486	rs1130409	rs1800975	rs149364215

Thermal conditions	(1) 95°C, 10 min (2) 92°C, 15 sec (3) 60°C, 1 min (4) Steps 2&3, 45×				

Dyes	ROX, HEX, FAM				

Ref dye	ROX				

**Table 2:** The distribution of genotypes and allele frequencies and the analysis of the odds ratio (OR) for 51Gln/His polymorphism of *APEX* gene in patients with colorectal cancer (CRC) and the control group.

Genotype/allele	Patients (*n* = 311)	Controls (*n* = 302^∗^)	OR (95% CI)	*p*
Gln/Gln	69	92	1 (ref)	—
*Gln/His*	*206*	*161*	*1.706 (1.174–2.480)*	*0.005*
His/His	36	49	0.979 (0.576–1.667)	0.920
Gln	344	345	1 (ref)	—
His	278	259	1.077 (0.859–1.349)	0.522

^∗^Genotype distribution in the Hardy-Weinberg equilibrium, *χ*^2^ = 0.125.

Values set in italics denote statistical significance.

**Table 3:** The distribution of genotypes and allele frequencies and the analysis of the odds ratio (OR) for 148Asp/Glu polymorphism of *APEX* gene in patients with colorectal cancer (CRC) and the control group.

Genotype/allele	Patients (*n* = 309)	Controls (*n* = 301^∗^)	OR (95% CI)	*p*
Asp/Asp	51	88	1 (ref)	—
*Asp/Glu*	*237*	*158*	*2.588 (1.736–3.859)*	*<0.0001*
Glu/Glu	21	55	0.659 (0.358–1.212)	0.179
Asp	339	334	1 (ref)	—
Glu	279	268	1.026 (0.819–1.285)	0.823

^∗^Genotype distribution in the Hardy-Weinberg equilibrium, *χ*^2^ = 0.277.

Values set in italics denote statistical significance.

**Table 4:** The distribution of genotypes and allele frequencies and the analysis of the odds ratio (OR) for 23Gly/Ala polymorphism of *XPA* gene in patients with colorectal cancer (CRC) and the control group.

Genotype/allele	Patients (*n* = 310)	Controls (*n* = 304^∗^)	OR (95% CI)	*p*
Gly/Gly	31	95	1 (ref)	—
*Gly/Ala*	*263*	*150*	*5.373 (3.418–8.446)*	*<0.0001*
Ala/Ala	16	59	0.831 (0.419–1.649)	0.597
Gly	325	340	1 (ref)	—
Ala	295	268	1.152 (0.920–1.442)	0.218

^∗^Genotype distribution in the Hardy-Weinberg equilibrium, *χ*^2^ = 0.988.

Values set in italics denote statistical significance.

**Table 5:** The distribution of genotypes and allele frequencies and the analysis of the odds ratio (OR) for 64Ile/Val polymorphism of *APEX* gene in patients with colorectal cancer (CRC) and the control group.

Genotype/allele	Patients (*n* = 307)	Controls (*n* = 304^∗^)	OR (95% CI)	*p*
Ile/Ile	93	78	1 (ref)	—
Ile/Val	148	159	0.781 (0.537–1.136)	0.195
Val/Val	66	67	0.826 (0.525–1.301)	0.409
Ile	334	315	1 (ref)	—
Val	280	293	0.901 (0.720–1.128)	0.365

^∗^Genotype distribution in the Hardy-Weinberg equilibrium, *χ*^2^ = 0.408.

**Table 6:** The distribution of genotypes and allele frequencies and the analysis of the odds ratio (OR) for 689Ser/Arg polymorphism of *ERCC4* gene in patients with colorectal cancer (CRC) and the control group.

Genotype/allele	Patients (*n* = 309)	Controls (*n* = 304^∗^)	OR (95% CI)	*p*
Ser/Ser	93	101	1 (ref)	—
Ser/Arg	155	160	1.052 (0.736–1.505)	0.777
Arg/Arg	61	43	1.541 (0.952–2.493)	0.078
Ser	341	362	1 (ref)	—
Arg	277	246	1.195 (0.953–1.499)	0.123

^∗^Genotype distribution in the Hardy-Weinberg equilibrium, *χ*^2^ = 0.107.

**Table 7:** The distribution of genotypes and the analysis of the odds ratio (OR) for gene-gene interactions in analyzed polymorphisms in patients with colorectal cancer (CRC) and the control group. Shown are only pairs that modulate the risk at a statistically significant level. All partial results for the gene-gene interaction are shown in the supplementary materials.

Gene-gene interaction	Genotype	Patients	Controls	OR (95% CI)	*p*
51Gln/His APEX-64Ile/Val APEX	Gln/His-Val/Val	55	32	2.266 (1.120–4.585)	0.022
51Gln/His APEX-148Asp/Glu APEX	Gln/His-Asp/Glu	174	102	2.003 (1.091–3.676)	0.023
	Gln/His-Glu/Glu	8	26	0.361 (0.137–0.951)	0.036
	His/His-Asp/Asp	6	28	0.252 (0.089–0.714)	0.007
51Gln/His APEX-23Gly/Ala XPA	Gln/His-Gly/Gly	6	35	0.220 (0.078–0.622)	0.003
	Gln/His-Gly/Ala	193	98	2.532 (1.362–4.707)	0.003
	His/His-Gly/Ala	30	8	4.821 (1.835–12.670)	0.001
51Gln/His APEX-689Ser/Arg ERCC4	Gln/His-Arg/Arg	39	24	2.464 (1.247–4.870)	0.009
64Ile/Val APEX-148Asp/Glu APEX	Ile/Val-Asp/Asp	10	27	0.403 (0.170–0.955)	0.036
	Val/Val-Asp/Glu	59	18	3.567 (1.765–7.211)	0.0003
64Ile/Val APEX-23Gly/Ala XPA	Ile/Val-Gly/Gly	9	37	0.269 (0.103–0.700)	0.006
	Ile/Val-Ala/Ala	6	23	0.288 (0.097–0.859)	0.022
	Val/Val-Gly/Ala	62	14	4.895 (2.093–11.445)	0.0001
148Asp/Glu APEX-23Gly/Ala XPA	Asp/Asp-Gly/Ala	26	58	0.399 (0.176–0.902)	0.025
	Asp/Glu-Gly/Gly	8	46	0.155 (0.056–0.424)	0.0001
	Asp/Glu-Gly/Ala	218	81	2.392 (1.164–4.915)	0.015
148Asp/Glu APEX-689Ser/Arg ERCC4	Asp/Asp-Ser/Arg	21	48	0.844 (0.370–1.924)	0.689
	Asp/Asp-Arg/Arg	13	13	1.929 (0.707–5.263)	0.198
	Asp/Glu-Ser/Ser	69	49	2.716 (1.293–5.704)	0.007
23Gly/Ala XPA-689Ser/Arg ERCC4	Gly/Ala-Ser/Ser	76	57	2.333 (1.061–5.131)	0.032
	Gly/Ala-Ser/Arg	135	77	3.068 (1.431–6.577)	0.003
	Gly/Ala-Arg/Arg	47	16	5.141 (2.073–12.749)	0.0003

**Table 8:** Analysis of correlation of selected polymorphisms with the state of tumor according to classification of American Joint Committee on Cancer.

51Gln/His polymorphism of APEX gene										
Genotype	Patients (*n* = 311)				II° versus I°		III° + IV° versus I°		III° + IV° versus II°	
	I°	II°	III°	IV°	OR (95% CI)	*p*	OR (95% CI)	*p*	OR (95% CI)	*p*
Gln/Gln	24	21	19	5	1 (ref)	—	1 (ref)	—	1 (ref)	—
Gln/His	57	94	48	7	1.885 (0.963–3.690)	0.062	0.965 (0.491–1.898)	0.920	0.512 (0.261–1.004)	0.049
His/His	19	15	2	0	0.902 (0.369–2.209)	0.823	—	—	—	—

64Ile/Val polymorphism of APEX										
Genotype	Patients (*n* = 307)				II° versus I°		III° + IV° versus I°		III° + IV° versus II°	
	I°	II°	III°	IV°	OR (95% CI)	*p*	OR (95% CI)	*p*	OR (95% CI)	*p*
Ile/Ile	41	32	19	1	1 (ref)	—	1 (ref)	—	1 (ref)	—
Ile/Val	64	51	28	5	1.021 (0.566–1.843)	1.000	1.057 (0.536–2.086)	0.862	1.035 (0.509–2.105)	0.920
Val/Val	33	17	14	2	0.660 (0.313–1.391)	0.273	0.994 (0.446–2.215)	1.000	1.506 (0.623–3.638)	0.362

148Asp/Glu polymorphism of APEX										
Genotype	Patients (*n* = 309)				II° versus I°		III° + IV° versus I°		III° + IV° versus II°	
	I°	II°	III°	IV°	OR (95% CI)	*p*	OR (95% CI)	*p*	OR (95% CI)	*p*
Asp/Asp	29	17	5	0	1 (ref)	—	1 (ref)	—	1 (ref)	—
Asp/Glu	98	133	5	1	*2.315 (1.205–4.449)*	*0.01*	—	—	—	—
Glu/Glu	16	5	0	0	0.533 (0.166–1.716)	0.288	—	—	—	—

23Gly/Ala polymorphism of XPA gene										
Genotype	Patients (*n* = 310)				II° versus I°		III° + IV° versus I°		III° + IV° versus II°	
	I°	II°	III°	IV°	OR (95% CI)	*p*	OR (95% CI)	*p*	OR (95% CI)	*p*
Gly/Gly	17	9	5	0	1 (ref)	—	1 (ref)	—	1 (ref)	—
Gly/Ala	87	165	9	2	*3.582 (1.533–8.370)*	*0.002*	—	—	—	—
Ala/Ala	11	5	0	0	0.859 (0.227–3.248)	0.823	—	—	—	—

689Ser/Arg polymorphism of ERCC4										
Genotype	Patients (*n* = 309)				II° versus I°		III° + IV° versus I°		III° + IV° versus II°	
	I°	II°	III°	IV°	OR (95% CI)	*p*	OR (95% CI)	*p*	OR (95% CI)	*p*
Ser/Ser	49	31	9	4	1 (ref)	—	1 (ref)	—	1 (ref)	—
Ser/Arg	83	52	16	4	0.990 (0.561–1.747)	1.000	0.908 (0.415–1.986)	0.806	0.795 (0.356–1.776)	0.578
Arg/Arg	32	24	3	2	1.186 (0.592–2.374)	0.632	0.589 (0.192–1.811)	0.351	0.431 (0.137–1.352)	0.143

Values set in italics denote statistical significance.
