# Supplementary material for: Modulation of Colorectal Cancer Risk by Polymorphisms in 51Gln/His, 64Ile/Val, and 148Asp/Glu of APEX Gene; 23Gly/Ala of XPA Gene; and 689Ser/Arg of ERCC4 Gene
Source: Gastroenterol Res Pract. 2017 Mar 12;2017:3840243. doi: 10.1155/2017/3840243 (PMC5366231; doi:10.1155/2017/3840243)
Supplement: Supplementary file 1 — Table 1. The distribution of genotypes and the analysis of the odds ratio (OR) for gene-gene interactions: 51Gln/His APEX – 64Ile/Val APEX in patients with colorectal cancer (CRC) and the control group. Table 2. The distribution of genotypes and the analysis of the odds ratio (OR) for gene-gene interactions: 51Gln/His APEX – 148Asp/Glu APEX in patients with colorectal cancer (CRC) and the control group. Table 3. The distribution of genotypes and the analysis of the odds ratio (OR) for gene-gene interactions: 51Gln/His APEX – 23Gly/Ala XPA in patients with colorectal cancer (CRC) and the control group. Table 4. The distribution of genotypes and the analysis of the odds ratio (OR) for gene-gene interactions: 51Gln/His APEX – 689Ser/Arg ERCC4 in patients with colorectal cancer (CRC) and the control group. Table 5. The distribution of genotypes and the analysis of the odds ratio (OR) for gene-gene interactions: 64Ile/Val APEX – 148Asp/Glu APEX in patients with colorectal cancer (CRC) and the control group. Table 6. The distribution of genotypes and the analysis of the odds ratio (OR) for gene-gene interactions: 64Ile/Val APEX – 23Gly/Ala XPA in patients with colorectal cancer (CRC) and the control group. Table 7. The distribution of genotypes and the analysis of the odds ratio (OR) for gene-gene interactions: 64Ile/Val APEX – 689Ser/Arg ERCC4 in patients with colorectal cancer (CRC) and the control group. Table 8. The distribution of genotypes and the analysis of the odds ratio (OR) for gene-gene interactions: 148Asp/Glu APEX – 23Gly/Ala XPA in patients with colorectal cancer (CRC) and the control group. Table 9. The distribution of genotypes and the analysis of the odds ratio (OR) for gene-gene interactions: 148Asp/Glu APEX – 689Ser/Arg ERCC4 in patients with colorectal cancer (CRC) and the control group. Table 10. The distribution of genotypes and the analysis of the odds ratio (OR) for gene-gene interactions: 23Gly/Ala XPA – 689Ser/Arg ERCC4 in patients with colorect [file 3840243.f1.pdf]

Table 1. The distribution of genotypes and the analysis of the odds ratio (OR) for gene-gene interactions: 51Gln/His *APEX* – 64Ile/Val *APEX* in patients with colorectal cancer (CRC) and the control group.

Genotype	Patients n=302	Controls n=302	OR (95% CI)	p
Gln/Gln-Ile/Ile	22	29	1 (ref)	-
Gln/Gln-Ile/Val	37	49	0.995 (0.495-2.003)	1.000
Gln/Gln-Val/Val	8	14	0.753 (0.269-2.111)	0.590
Gln/His-Ile/Ile	61	42	1.915 (0.971-3.777)	0.059
Gln/His-Ile/Val	84	87	1.273 (0.678-2.390)	0.454
**Gln/His-Val/Val**	**55**	**32**	**2.266 (1.120-4.585)**	**0.022**
His/His-Ile/Ile	9	6	1.977 (0.612-6.385)	0.251
His/His-Ile/Val	25	22	1.498 (0.675-3.324)	0.320
His/His-Val/Val	1	21	-	-

Table 2. The distribution of genotypes and the analysis of the odds ratio (OR) for gene-gene interactions: 51Gln/His *APEX* – 148Asp/Glu *APEX* in patients with colorectal cancer (CRC) and the control group.

Genotype	Patients n=301	Controls n=301	OR (95% CI)	p
Gln/Gln-Asp/Asp	23	27	1 (ref)	-
Gln/Gln-Asp/Glu	39	54	0.848 (0.424-1.694)	0.639
Gln/Gln-Glu/Glu	3	10	-	-
Gln/His-Asp/Asp	19	33	0.676 (0.306-1.493)	0.332
**Gln/His-Asp/Glu**	**174**	**102**	**2.003 (1.091-3.676)**	**0.023**
**Gln/His-Glu/Glu**	**8**	**26**	**0.361 (0.137-0.951)**	**0.036**
**His/His-Asp/Asp**	**6**	**28**	**0.252 (0.089-0.714)**	**0.007**
His/His-Asp/Glu	20	2	-	-
His/His-Glu/Glu	9	19	0.556 (0.211-1.465)	0.233

Table 3. The distribution of genotypes and the analysis of the odds ratio (OR) for gene-gene interactions: 51Gln/His *APEX* – 23Gly/Ala *XPA* in patients with colorectal cancer (CRC) and the control group.

Genotype	Patients n=302	Controls n=302	OR (95% CI)	p
Gln/Gln-Gly/Gly	21	27	1 (ref)	-
Gln/Gln-Gly/Ala	35	43	1.047 (0.507-2.159)	0.888
Gln/Gln-Ala/Ala	9	22	0.526 (0.201-1.377)	0.188
**Gln/His-Gly/Gly**	**6**	**35**	**0.220 (0.078-0.622)**	**0.003**
**Gln/His-Gly/Ala**	**193**	**98**	**2.532 (1.362-4.707)**	**0.003**
Gln/His-Ala/Ala	4	28	-	-
His/His-Gly/Gly	2	32	-	-
**His/His-Gly/Ala**	**30**	**8**	**4.821 (1.835-12.670)**	**0.001**
His/His-Ala/Ala	2	9	-	-

Table 4. The distribution of genotypes and the analysis of the odds ratio (OR) for gene-gene interactions: 51Gln/His *APEX* – 689Ser/Arg *ERCC4* in patients with colorectal cancer (CRC) and the control group.

Genotype	Patients n=302	Controls n=302	OR (95% CI)	p
Gln/Gln-Ser/Ser	31	47	1 (ref)	-
Gln/Gln-Ser/Arg	24	36	1.011 (0.508-2.010)	1.000
Gln/Gln-Arg/Arg	12	9	2.022 (0.762-5.365)	0.153
Gln/His-Ser/Ser	37	29	1.934 (0.995-3.761)	0.051
Gln/His-Ser/Arg	123	108	1.340 (0.994-1.806)	0.039
**Gln/His-Arg/Arg**	**39**	**24**	**2.464 (1.247-4.870)**	**0.009**
His/His-Ser/Ser	22	23	1.450 (0.692-3.039)	0.325
His/His-Ser/Arg	6	16	0.569 (0.201-1.612)	0.286
His/His-Arg/Arg	8	10	1.213 (0.431-3.413)	0.718

Table 5. The distribution of genotypes and the analysis of the odds ratio (OR) for gene-gene interactions: 64Ile/Val *APEX* – 148Asp/Glu *APEX* in patients with colorectal cancer (CRC) and the control group.

Genotype	Patients n=301	Controls n=301	OR (95% CI)	p
Ile/Ile-Asp/Asp	34	37	1 (ref)	-
Ile/Ile-Asp/Glu	47	29	1.764 (0.915-3.401)	0.089
Ile/Ile-Glu/Glu	11	11	1.088 (0.418-2.833)	0.862
**Ile/Val-Asp/Asp**	**10**	**27**	**0.403 (0.170-0.955)**	**0.036**
Ile/Val-Asp/Glu	126	111	1.235 (0.726-2.101)	0.435
Ile/Val-Glu/Glu	9	20	0.490 (0.196-1.222)	0.122
Val/Val-Asp/Asp	4	24	-	-
**Val/Val-Asp/Glu**	**59**	**18**	**3.567 (1.765-7.211)**	**0.0003**
Val/Val-Glu/Glu	1	24	-	-

Table 6. The distribution of genotypes and the analysis of the odds ratio (OR) for gene-gene interactions: 64Ile/Val *APEX* – 23Gly/Ala *XPA* in patients with colorectal cancer (CRC) and the control group.

Genotype	Patients n=304	Controls n=304	OR (95% CI)	p
Ile/Ile-Gly/Gly	19	21	1 (ref)	-
Ile/Ile-Gly/Ala	64	37	1.912 (0.911-4.011)	0.084
Ile/Ile-Ala/Ala	8	20	0.442 (0.158-1.236)	0.117
**Ile/Val-Gly/Gly**	**9**	**37**	**0.269 (0.103-0.700)**	**0.006**
Ile/Val-Gly/Ala	132	99	1.474 (0.752-2.889)	0.258
**Ile/Val-Ala/Ala**	**6**	**23**	**0.288 (0.097-0.859)**	**0.022**
Val/Val-Gly/Gly	2	37	-	-
**Val/Val-Gly/Ala**	**62**	**14**	**4.895 (2.093-11.445)**	**0.0001**
Val/Val-Ala/Ala	2	16	-	-

Table 7. The distribution of genotypes and the analysis of the odds ratio (OR) for gene-gene interactions: 64Ile/Val *APEX* – 689Ser/Arg *ERCC4* in patients with colorectal cancer (CRC) and the control group.

Genotype	Patients n=304	Controls n=304	OR (95% CI)	p
Ile/Ile-Ser/Ser	29	33	1 (ref)	-
Ile/Ile-Ser/Arg	43	34	1.439 (0.735-2.817)	0.288
Ile/Ile-Arg/Arg	19	11	1.966 (0.803-4.808)	0.136
Ile/Val-Ser/Ser	38	34	1.272 (0.644-2.511)	0.488
Ile/Val-Ser/Arg	88	107	0.936 (0.528-1.660)	0.823
Ile/Val-Arg/Arg	21	18	1.328 (0.595-2.964)	0.488
Val/Val-Ser/Ser	25	34	0.837 (0.408-1.716)	0.624
Val/Val-Ser/Arg	21	19	1.258 (0.567-2.789)	0.572
Val/Val-Arg/Arg	20	14	1.626 (0.698-3.787)	0.258

Table 8. The distribution of genotypes and the analysis of the odds ratio (OR) for gene-gene interactions: 148Asp/Glu *APEX* – 23Gly/Ala *XPA* in patients with colorectal cancer (CRC) and the control group.

Genotype	Patients n=301	Controls n=301	OR (95% CI)	p
Asp/Asp-Gly/Gly	18	16	1 (ref)	-
**Asp/Asp-Gly/Ala**	**26**	**58**	**0.399 (0.176-0.902)**	**0.025**
Asp/Asp-Ala/Ala	6	14	0.381 (0.118-1.227)	0.102
**Asp/Glu-Gly/Gly**	**8**	**46**	**0.155 (0.056-0.424)**	**0.0001**
**Asp/Glu-Gly/Ala**	**218**	**81**	**2.392 (1.164-4.915)**	**0.015**
Asp/Glu-Ala/Ala	4	31	-	-
Glu/Glu-Gly/Gly	4	32	-	-
Glu/Glu-Gly/Ala	11	9	1.086 (0.358-3.293)	0.888
Glu/Glu-Ala/Ala	6	14	0.381 (0.118-1.227)	0.102

Table 9. The distribution of genotypes and the analysis of the odds ratio (OR) for gene-gene interactions: 148Asp/Glu *APEX* – 689Ser/Arg *ERCC4* in patients with colorectal cancer (CRC) and the control group.

Genotype	Patients n=301	Controls n=301	OR (95% CI)	p
Asp/Asp-Ser/Ser	14	27	1 (ref)	-
Asp/Asp-Ser/Arg	21	48	0.844 (0.370-1.924)	0.689
Asp/Asp-Arg/Arg	13	13	1.929 (0.707-5.263)	0.198
**Asp/Glu-Ser/Ser**	**69**	**49**	**2.716 (1.293-5.704)**	**0.007**
**Asp/Glu-Ser/Arg**	**126**	**82**	**2.963 (1.467-5.985)**	**0.002**
**Asp/Glu-Arg/Arg**	**37**	**27**	**2.643 (1.171-5.965)**	**0.018**
Glu/Glu-Ser/Ser	8	25	0.617 (0.221-1.720)	0.354
Glu/Glu-Ser/Arg	4	27	-	-
Glu/Glu-Arg/Arg	9	3	-	-

Table 10. The distribution of genotypes and the analysis of the odds ratio (OR) for gene-gene interactions: 23Gly/Ala *XPA* – 689Ser/Arg *ERCC4* in patients with colorectal cancer (CRC) and the control group.

Genotype	Patients n=304	Controls n=304	OR (95% CI)	p
Gly/Gly-Ser/Ser	12	21	1 (ref)	-
Gly/Gly-Ser/Arg	13	49	0.464 (0.182-1.184)	0.105
Gly/Gly-Arg/Arg	6	25	0.420 (0.135-1.312)	0.130
**Gly/Ala-Ser/Ser**	**76**	**57**	**2.333 (1.061-5.131)**	**0.032**
**Gly/Ala-Ser/Arg**	**135**	**77**	**3.068 (1.431-6.577)**	**0.003**
**Gly/Ala-Arg/Arg**	**47**	**16**	**5.141 (2.073-12.749)**	**0.0003**
Ala/Ala-Ser/Ser	4	23	-	-
Ala/Ala-Ser/Arg	3	34	-	-
Ala/Ala-Arg/Arg	8	2	-	-
